# Biosynthesis of Silver Nanoparticles Using *Commiphora mukul* Extract: Evaluation of Anti-Arthritic Activity in Adjuvant-Induced Arthritis Rat Model

**DOI:** 10.3390/pharmaceutics14112318

**Published:** 2022-10-28

**Authors:** Anupama Singh, Sateesha Shivally Boregowda, Afrasim Moin, Amr Selim Abu Lila, Mohammed F. Aldawsari, El-Sayed Khafagy, Hadil Faris Alotaibi, Rajamma Abburu Jayaramu

**Affiliations:** 1 Department of Pharmaceutics, Acharya & BM Reddy College of Pharmacy, Bengaluru 560090, India; 2 Department of Pharmaceutics, College of Pharmacy, University of Hail, Hail 81442, Saudi Arabia; 3 Department of Pharmaceutics and Industrial Pharmacy, Faculty of Pharmacy, Zagazig University, Zagazig 44519, Egypt; 4 Department of Pharmaceutics, College of Pharmacy, Prince Sattam Bin Abdulaziz University, Al-Kharj 11942, Saudi Arabia; 5 Department of Pharmaceutics and Industrial Pharmacy, Faculty of Pharmacy, Suez Canal University, Ismailia 41522, Egypt; 6 Department of Pharmaceutical Sciences, College of Pharmacy, Princess Nourah bint Abdulrahman University, Riyadh 11671, Saudi Arabia; 7 Department of Pharmacognosy, KLE College of Pharmacy, Bengaluru 560010, India

**Keywords:** anti-inflammatory, arthritic score, guggul, rheumatoid arthritis, silver nanoparticles

## Abstract

Rheumatoid arthritis (RA) is a major global public health challenge, and novel therapies are required to combat it. Silver nanoparticles (AgNPs) have been employed as delivery vehicles of anti-inflammatory drugs for RA therapy, and it has been recently realized that AgNPs have anti-inflammatory action on their own. However, their conventional synthesis processes might result in cytotoxicity and environmental hazards. Instead, the use of natural products as a reducing and stabilizing agent in the biosynthesis of silver nanoparticles has arisen as an option to decrease the cytotoxic and environmental concerns associated with chemical synthesis of AgNPs. In this study, we challenged the efficacy of *Commiphora mukul* (guggul) aqueous extract as a reducing and/or capping agent for the biosynthesis of AgNPs. Guggul-mediated biosynthesized silver nanoparticles (G-AgNPs) were characterized via UV-vis spectroscopy, dynamic light scattering, and scanning electron microscopy. In addition, their anti-arthritic potential was evaluated in an adjuvant-induced arthritis (AIA) model. The fabricated NPs showed an absorption peak at 412 nm, corresponding to the typical surface plasmon resonance band of AgNPs. The synthesized G-AgNPs were nearly spherical, with a particle size of 337.6 ± 12.1 nm and a negative surface charge (−18.9 ± 1.8 mV). In AIA rat model, synthesized G-AgNPs exerted a potent anti-inflammatory action, as manifested by a remarkable reduction in paw volume (>40%) along with elicitation of a minimal arthritic score, compared to control rats. In addition, when compared to arthritic rats, treatment with G-AgNPs efficiently restored the activity of antioxidant enzyme, superoxide dismutase, and catalase, indicating the efficiency of synthesized G-AgNPs in alleviating the oxidative stress associated with RA. Finally, histological examination revealed comparatively lower inflammatory cells infiltration in ankle joint tissue upon treatment with G-AgNPs. Collectively, biosynthesized G-AgNPs might represent a plausible therapeutic option for the management of RA.

## 1. Introduction

Rheumatoid arthritis (RA) is an autoimmune disease with a global prevalence of 1% [1]. It is defined by chronic joint inflammation, large infiltration of activated immune cells, and bone or cartilage damage [2]. The pathophysiology of RA is still unclear, but multiple mechanistic investigations have shown that an influx of activated inflammatory B cells, T cells, and macrophages produce a variety of pro-inflammatory cytokines that lead to severe tissue damage and joint destruction [1,3]. Currently, there are three classes of pharmaceuticals that are routinely used in clinic for the management of RA, including glucocorticoids, nonsteroidal anti-inflammatory drugs, and disease-modifying anti-rheumatic drugs. Nevertheless, despite the fact that these treatment modalities can alleviate symptoms and halt the course of arthritis, high doses and frequent administration are frequently necessary to achieve sufficient effectiveness, which ultimately results in unfavorable side effects [4,5]. Consequently, innovative therapeutic approaches for RA therapy are still desperately needed.

In recent years, there has been a surge in the development of nanomedicine for treating challenging inflammatory diseases, including RA, with the goal of avoiding the shortcomings associated with conventional therapies. The unique pathophysiology of RA, including the leaky blood vessels and inflammatory cell infiltration, efficiently augmented the passive and preferential accumulation of nanocarriers via what is known as the ELVIS effect (extravasation through leaky vasculature and inflammatory cell-mediated sequestration) at the disease site [6]. Furthermore, the overexpression of particular receptors on the cell membrane of inflammation-associated cells triggered the development of many actively targeted drug delivery systems, via modifying their surface with certain recognition ligands [7,8,9,10,11,12]. Several nanoparticulate systems have been recently studied in anti-inflammatory treatment [13,14,15]. For instance, Kuskov et al. [16] emphasized the utility of amphiphilic poly-*N*-vinylpyrrolidone (PVP) nanoparticles as carriers for nonsteroidal anti-inflammatory drug, indomethacin. They reported that orally administered indomethacin-loaded PVP nanoparticles displayed superior in vivo anti-inflammatory efficacy with less ulcerogenic potential, in carrageenan-induced paw edema animal model, compared to free indomethacin. Alaaeldin et al. [15] have demonstrated the efficacy of spanlastic nanovesiclular system for ameliorating the anti-inflammatory effect of the selective COX-2 inhibitor, celecoxib, in an adjuvant-induced arthritis (AIA) rat model, via suppression of the proinflammatory markers, tumor necrosis factor alpha (TNF-α), and nuclear factor kappa-B (NFкB). In the same context, Singh et al. [1] have recently underscored the potential of intra-articularly administered poly ε-carpolactone nanoparticles loaded with the immunosuppressive disease-modifying antirheumatic drug, leflunomide, for alleviating serum levels of proinflammatory markers and inflammatory cytokines, with subsequent reduction in paw volume and knee diameter in a AIA rodent model.

Metal nanoparticles have recently sparked increased interest for medicinal and diagnostic applications. Their unique biological and/or physicochemical properties verify their drug delivery efficiency [17,18,19]. Among them, silver nanoparticles (AgNPs) are favored for constructing drug nano-delivery vehicles, owing to their ease of synthesis and modification and diverse biological activities [20,21]. Furthermore, because of their efficient cross-membrane transport, they can readily deliver their payload into the cells. Recently, silver nanoparticles have been employed as carriers to deliver anti-inflammatory medicines for the treatment of RA [22,23].

Silver nanoparticles (AgNPs) are commonly produced and utilized chemical-reducing agents that might exert negative consequences on human health and the biological application of nanoparticles. Instead, green chemistry has recently gained popularity in the synthesis of metal nanoparticles. It synthesizes metal nanoparticles in a more eco-friendly, non-toxic, cost-effective, and rapid process [24,25]. Such a biological approach involves using either micro-organisms or plants for their synthesis [26]. The use of plant extracts as reducing and stabilizing agents to synthesize AgNPs offers the merits of low cost, eco-friendliness, and ease of operation. Aside from this, the phytoconstituents themselves used for the synthesis of nanoparticles might synergistically augment the therapeutic effect of the synthesized AgNPs [21].

Guggul (*Commiphora mukul*) is a resin extract that has been used in Ayurvedic medicine for centuries for the treatment of various conditions, including obesity, skin diseases, and arthritis [27]. The oleo gum resin of *Commiphora mukul* (Guggul) is rich in phytoconstituents, such as terpenoids, steroids, and lignans [28], which can act as powerful reducing agents; they have been reported to exert potent anti-inflammatory activity in various animal models [29]. Interestingly, the anti-inflammatory effect of guggul was found to be comparable to that of ibuprofen, phenylbutazone, and hydrocortisone [27]. The aim of this study, therefore, was to green-synthesize AgNPs using guggul as a reducing agent. In addition, the therapeutic efficacy of Guggul-mediated synthesized silver nanoparticles (G-AgNPs) in controlling the inflammation was assessed in an adjuvant-induced arthritis rat model.

## 2. Materials and Methods

### 2.1. Materials

Silver nitrate, Complete Freund’s adjuvant, and methotrexate was purchased from Sigma Aldrich-Merck Ltd. (Mumbai, India). *Commiphora mukul* (oleo gum resin) was provided from Research Lab Fine Chem Industry (Mumbai, India). All other chemicals and reagents used in the research were of analytical grade.

### 2.2. Preparation of Commiphora mukul Plant (Guggul) Extract

The oleo gum resin was converted into a fine powder using mortar and pestle. Briefly, 10% (*w*/*v*) oleo gum resin extract was prepared in double distilled water following the decoction process, to obtain the bioactive compound [30]. The extract was then filtered using Whatman filter paper and centrifuged at 3000 rpm for 30 min to remove very fine particles and to obtain a clear extract. The phytoconstituents of the extract were determined using different phytochemical tests (Table S1). The final extract was stored in the refrigerator at 2–8 °C for further use.

### 2.3. Synthesis of Silver Nanoparticles (AgNPs)

The green synthesis of AgNPs was conducted, using guggul extract as a reducing and stabilizing agent. Initially, various concentrations of guggul extract (20–100 mg/mL) were mixed with silver nitrate solution (1–5 mM) in 1:9 *v*/*v* ratio under continuous stirring at the temperature of 80 ± 5 °C. The reaction media was maintained at alkaline pH to favor the reaction and stability of the nanoparticles. The color change of reaction mixture from light yellow to dark brown indicated the synthesis of G-AgNPs. The synthesized nanoparticles were then centrifuged (Cooling Centrifuge, REMI, Mumbai, India) at 5000 rpm for 30 min at 25 °C. Afterwards, the sedimented pellets obtained were re-dispersed in deionized water, washed thrice, and re-centrifuged. Finally, the obtained pellets were lyophilized (Labconco, MO, USA) at −80 °C overnight and stored for future studies [31].

### 2.4. Characterization of Silver Nanoparticles

#### 2.4.1. UV Spectroscopy Analysis

Silver nanoparticles synthesized using guggul extract were analyzed for their purity using UV-Vis Spectroscopy. Briefly, a small aliquot of the reaction mixtures was taken off and diluted with sterile distilled water (1:10). The UV-Vis. spectrum was then recorded at a wavelength from 300 to 800 nm using UV Spectrophotometer (Agilent Cary 60 UV-Vis, Santa Clara, CA, USA).

#### 2.4.2. Particle Size Measurement

The mean particle size of the formed nanoparticles was determined using a zeta sizer (SZ-100, Horiba Scientific, Kyoto, Japan). Briefly, 1 mL of the sample was diluted 10 times and placed in a cuvette for the analysis of particle size and size distribution [32].

#### 2.4.3. Surface Charge Determination

Zeta potential was measured for the surface charge. The nanoparticles were diluted 10 times with deionized water and taken in an electrode cuvette for the measurement of zeta potential using a zeta sizer instrument (SZ-100, Horiba Scientific, Kyoto, Japan) [18].

#### 2.4.4. Surface Morphology

Scanning electron microscopy (SEM) is employed to understand the surface topographical properties, such as size, shape, texture, and porosity, of the silver nanoparticles. The SEM of the particles was captured using ZEISS Scanning Electron Microscopy (CAREL ZEISS, EVO 18, Jena, Germany). The SEM images of silver nanoparticles were taken at a magnification of 50,000×.

#### 2.4.5. Fourier Transform Infrared (FT-IR)

The stability of nanoparticles is an important criterion. To recognize the bioactive molecules capping over the synthesized nanoparticles, FT-IR analysis was conducted for both guggul-capped silver nanoparticles and guggul extract using Bruker FT-IR instrument (Billerica, MA, USA). The FT-IR spectra were recorded within the wave number range 4000 cm^−1^–400 cm^−1^ [33].

### 2.5. In-Vivo Anti-Arthritic Activity

#### 2.5.1. Animal Study

Female Wistar albino rats (6–5 weeks old, 180–200 g) were used in this study. The animals were provided with free access to water and food and were housed in cages with a 12-h dark/light cycle under standardized temperature (21–25 °C) and relative humidity (50–70%) conditions. All experimental protocols were reviewed and approved by the Institutional Animal Ethics Committee of Acharya and BM Reddy College of Pharmacy, Bengaluru, India (Approval number: IAEC/ABMRCP/2020-21/03). General and environmental conditions were monitored as per Organization for Economic Co-operation and Development (OECD) guidelines.

#### 2.5.2. Acute Toxicity Study

The acute oral toxicity for the silver nanoparticles was carried out as per the OECD Test Guidelines 425 [34]. Briefly, animals were fasted for 3–4 h before treatment, but given free access to water. The limit test was carried out at a dose of 2000 mg/kg p.o. as a single dose, given to a single rat; the animal was closely monitored over a period of 14 days for mortality and physical/behavior changes. Afterwards, a group of rats was fed orally with silver nanoparticles at a minimum dose of 1.75 mg/kg and were observed for 14 days. Finally, based on mortality results, the rats were treated with much lower doses of silver nanoparticles (100, 200 and 300 µg/kg) and observed for 7 days.

#### 2.5.3. Adjuvant-Induced Arthritis Rodent Model

The adjuvant-induced arthritis (AIA) model is one of the most frequently adopted standard arthritis models [35]. To induce arthritis, rats were injected into the sub-plantar surface of the right hind paw with 0.1 mL of Freund’s Complete Adjuvant Emulsion (CFA). After 1 week of administration of CFA (incubation period), the rats were divided into four groups, with six rats in each group. The first group (non-arthritic rats) received normal saline (1 mL/kg, p.o) and served as a negative control. The second group (arthritic control) received normal saline (1 mL/kg/day, p.o) and served as a positive control. The third group comprised arthritic rats treated with the test dose of silver nanoparticles (determined after toxicity study, i.e., 200 µg/kg, p.o.) on a daily basis for a period of 14 days. The last group involved arthritic rats treated intraperitoneally with methotrexate (0.4 mg/kg/day) and served as a standard group. The antiarthritic efficiency was evaluated in terms of change of body weight, paw volume and arthritic score. Body weight was measured on weekly basis, whereas paw volume was measured at regular short intervals using a digital plethysmometer. The arthritic score, which reflects the severity and degree of inflammation in each limb of the rat, was graded by the visual criteria method using a digital plethysmometer. The score was set at a scale of 0–4, where 0 equals “no inflammation”, 1 equals “mild inflammation of 1 joint”, 2 equals “moderate inflammation of 1 joint”, 3 equals “severe inflammation of 1 joint”, and 4 equals “maximum inflammation of more than one joint in the limb”. The arthritic index was recorded as a total score of 16 per animal (score of 4 for each limb) [36].

#### 2.5.4. Radiological Assay

For radiological assay, rats were anesthetized by sodium thiopental (40 mg/kg, i.p.). The rats were then placed on an X-ray plate and imaged at a 25 cm focus to film distance to assess soft tissue swelling, joint space narrowing (cartilage loss), bone degeneration (erosions), and articular surface regularity [37].

#### 2.5.5. Estimation of Antioxidant Activity

##### Estimation of Superoxide Dismutase

Paw tissue was homogenized in an ice-cold tris-buffer with a homogenizer. The homogenate was centrifuged, and the supernatant was subjected to superoxide dismutase (SOD) estimation. Briefly, 0.1 mL aliquot of the supernatant was mixed with 1.2 mL 0.052 M sodium pyrophosphate buffer, followed by 0.1 mL of 186 µM phenazine emethosulphate, 0.3 mL of 300 µM Nitro blue tetrazolium, and 0.2 mL of 780 µM nicotinamide adenine dinucleotide hydrogen (NADH). The reaction was halted by adding 1 mL of glacial acetic acid to the reaction mixture and incubating it at 30 °C for 90 min. The reaction mixture was agitated, mixed with 4 mL of n-butanol, and then centrifuged for 10 min at 4000 rpm. The absorbance of the organic layer was then measured at 550 nm using a spectrophotometer. As a blank, 0.1 mL of distilled water was used instead of paw homogenate. The enzymatic activity is defined as the enzyme concentration (U/mg) required to reduce the absorbance of chromogen by 50% in the control sample under test conditions. The enzymatic activity was estimated using the following formula [38]:

$$\mathrm{SOD}\;\mathrm{activity}\;=\frac{({\mathrm{Absorbance}}_{\mathrm{control}}-{\;\mathrm{Absorbance}}_{\mathrm{Sample}})\;\left(V_{\mathrm{reaction}\;\mathrm{total}}\right)}{0.5\;({\mathrm{Absorbance}}_{\mathrm{control}})\;\left(V_{\mathrm{sample}}\right)}\times \;\mathrm{dilution}\;\mathrm{factor}$$

##### Estimation of Catalase (CAT)

Paw tissue was homogenized in an ice-cold phosphate buffer with a homogenizer. The homogenate was centrifuged, and the supernatant was subjected to catalase (CAT) estimation. Briefly, in a cuvette, 1.9 mL of 50 mM phosphate buffer was added to 0.1 mL of supernatant of paw tissue homogenate. To this mixture, 1.0 mL of freshly made 30 mM H2O2 was added, and UV absorbance was measured at 240 nm using spectrophotometer for 3 min at intervals of 30 s. A control sample was prepared using 0.1 mL distilled water instead of tissue homogenate. Catalase activity is expressed as the amount of enzyme (U/mg) required to prevent a 50% change in absorbance in one minute in the control sample [39]. The enzymatic activity was estimated using the following formula:

$$\mathrm{CAT}\;\mathrm{activity}=\frac{\mathrm{Absorbance}\;\times \;\mathrm{Volume}\;\mathrm{of}\;\mathrm{reaction}\;\mathrm{mixture}}{43.6\times \;\mathrm{Volume}\;\mathrm{of}\;\mathrm{sample}}\times \frac{1}{\mathrm{mg}\;\mathrm{of}\;\mathrm{protein}}$$

#### 2.5.6. Histopathology Assessment

At the end of in vivo experiments, rats were euthanized, and the right hind paw was excised, fixed in 10% formalin, and processed to make a paraffin block. Finally, 5 μm sections were stained with haematoxylin and eosin (H&E) staining for histological assessment.

### 2.6. Statistical Analysis

The results were expressed as Mean ± SEM (*n* = 6). Paw volume, arthritic score, body weight, and antioxidant parameters were analyzed using one-way ANOVA. Individual groups were compared against the control group using post-hoc Dunnet’s test. A *p* < 0.05 was considered to be statistically significant.

## 3. Results

### 3.1. Phytochemical Analysis of Commiphora mukul

Previous reports have emphasized that the oleo gum resin of *Commiphora mukul* (Guggul) is rich in phytoconstituents, such as terpenoids, steroids, and lignans [28]. In this study, qualitative analysis of the guggul extract (Table S1) confirmed the presence of terpenoids and steroidal phytoconstituents in the aqueous extract of *Commiphora mukul*.

### 3.2. Synthesis of Silver Nanoparticles

The green synthesis of silver nanoparticles was conducted using an aqueous extract of dried oleo gum resin of *Commiphora mukul* (Guggul). In this approach, guggul was exploited as a reducing and stabilizing agent as well. The formation of nanoparticles was confirmed by visual observation of the transformation of yellowish silver nitrate solution to dark brown (silver nanoparticles) within 20 min of the reaction. The presence of terpenoids, confirmed by Salkowski’s test, emphasized the potential of guggul to act as an efficient reducing agent for the synthesis of stable silver nanoparticles [40]. It was proposed that the phytoconstituents in guggul (in particular, terpenoids) interacted with the silver nitrate molecules and reduce them from the bulk. After the reduction, the atomic silver coalesces from atomic size to nanoparticle size via a process known as nucleation. Eventually, the bioactive molecules interact with the aggregated metal atoms and get adsorbed onto the surface, improving the thermodynamic stability of nanoparticles and making them an ideal therapeutic moiety.

### 3.3. Study of Process Parameters on Nanoparticle Formation

Nanoparticle formulations are greatly affected by different processing parameters, such as pH, the concentration of silver ions, and the concentration of reducing agents [23]. Accordingly, preliminary experiments were conducted to study the impact of various process parameters, including pH, concentration of silver nitrate, and concentration of guggul extract on nanoparticle formation. The formation of AgNPs was confirmed by the appearance of a distinct peak in the UV spectrum at the region of 400–800 nm, resulting from surface plasmon resonance (SPR), a characteristic phenomenon to noble metal nanoparticles [24].

#### 3.3.1. Influence of pH on the Nanoparticle Formation

**Figure 1:**
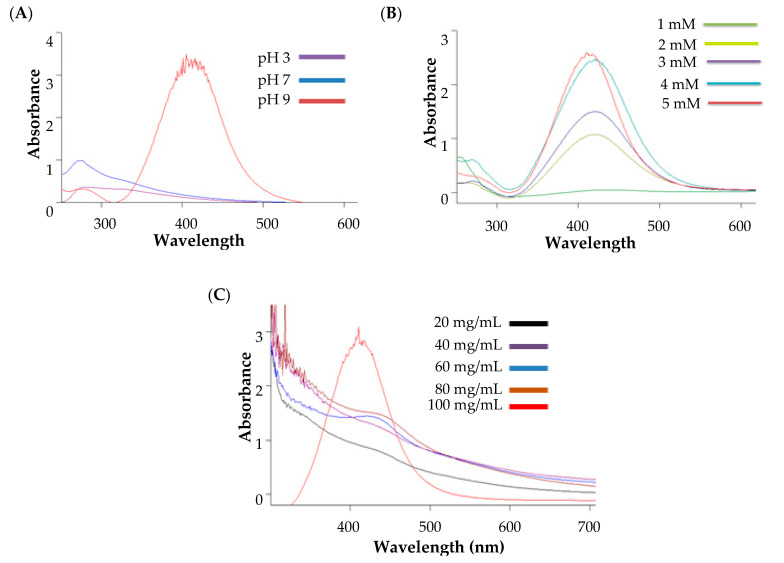
UV–visible spectra showing effect of (**A**) pH; (**B**) silver nitrate concentration; and (**C**) guggul concentration on the synthesis of AgNPs.

Three trials were conducted at three different pH values (pH 3, pH 7 and pH 9). As shown in Figure 1A, no nanoparticles were formed at both acidic (pH 3) and neutral (pH 7) conditions, as manifested by the lack of color change of the medium and the absence of SPR peak. On the other hand, alkaline pH (pH 9) was found to favor the formation of silver nanoparticles, as indicated by an obvious color change to dark brown/black and the appearance of a SPR peak at around 420 nm (Figure 1A). These results suggest that the synthesis of AgNPs was supported in an alkaline pH, rather than an acidic or neutral pH. Both an acidic and neutral pH might hinder the protonation and deprotonation mechanism [41]; thereby, no reduction of metal into metal nanoparticles would be favored.

#### 3.3.2. Influence of Silver Salt Concentration on the Nanoparticle Formation

In order to investigate the effect of silver salt concentration on nanoparticle formation, different silver salt concentrations, ranging from 1 mM to 5 mM, were used for the synthesis of AgNPs. As depicted in Figure 1B, low silver salt concentration (1 mM) failed to trigger the formation of silver nanoparticles by guggul. By contrary, sequential increases in the silver concentration, from 2 mM to 5 mM, resulted in a remarkable concentration-dependent increase in the absorption behavior of synthesized AgNPs (Figure 1B).

#### 3.3.3. Influence of Guggul Extract on the Nanoparticle Formation

The concentration of guggul is considered crucial for the synthesis of silver nanoparticles. Variation in guggul extract concentration from 20 mg/mL to 100 mg/mL in the reaction mixture of 5 mM silver was tested. As shown in Figure 1C, at lower extract concentrations (20 mg/mL to 80 mg/mL), guggul was not capable of bio-reducing silver salt into silver nanoparticles, as indicated by the absence of characteristic peaks in the SPR spectrum. On the other hand, a higher concentration of guggul (100 mg/mL) efficiently achieved the formation of AgNPs, as manifested by the existence of a characteristic SPR peak (Figure 1C). These results might be ascribed to the fact that at, higher guggul concentrations, the number of reducing groups increases in the reaction mixture to the optimal level, which is required to trigger the conversion of silver nitrate salt to AgNPs.

#### 3.3.4. Optimized Process for Nanoparticle Preparation

Based on our preliminary experiments, the optimal process conditions for guggul-mediated synthesis of silver nanoparticles include mixing silver salt (5 mM) with guggul extract (100 mg/mL) at an alkaline condition (pH 9).

### 3.4. Characterization of Silver Nanoparticles

#### 3.4.1. UV Spectroscopy Analysis

**Figure 2:**
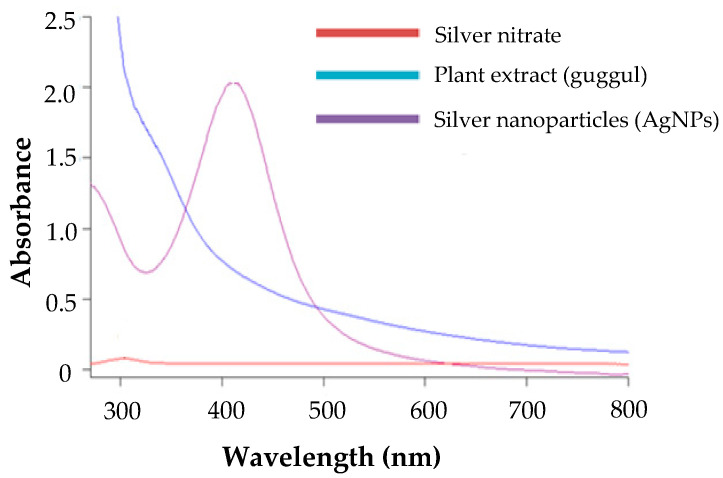
Surface Plasmon Resonance (SPR) depicted by UV Spectroscopy of silver nanoparticles.

The optical property of metal nanoparticles is a unique feature in their characterization. Surface Plasmons resonance (SPR) is the most spectacular optical property of metallic nanostructures. It indicates the appearance of a resonance effect caused by the interaction of metal nanoparticle conduction electrons with light photons [42]. This interaction is influenced by the shape and size of the metal nanoparticles, as well as the composition and nature of the dispersion medium [43]. It has been reported that tiny spherical silver nanoparticles have an SPR band that extends from 350 to 500 nm, with a peak position around 410 nm [44,45]. Similarly, in this study, the silver nanoparticles showed a characteristic absorbance peak at 412 nm (Figure 2), indicating the successful synthesis of G-AgNPs. Of note, such a peak was absent in the individual spectra of plant extract and silver nitrate salt, suggesting that, when silver ions are reduced into the nanoparticle range, a characteristic absorbance peak will appear, due to the optical SPR properties of the formed nanoparticles.

#### 3.4.2. Surface Morphology

**Figure 3:**
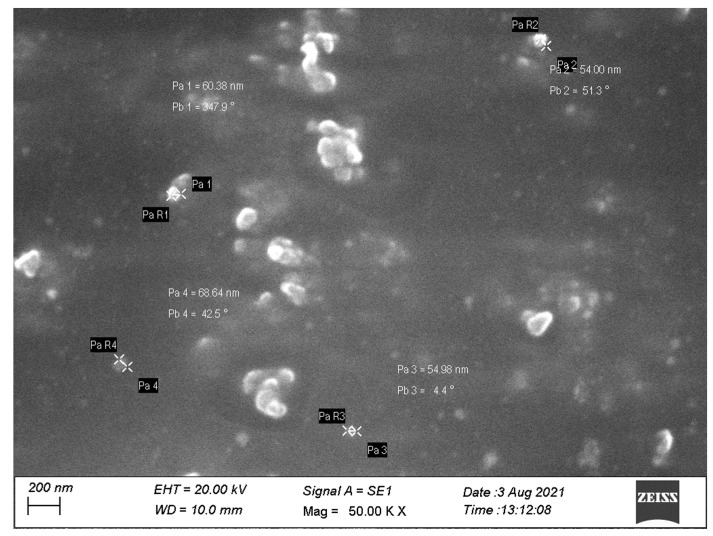
Surface morphology of silver nanoparticles.

SEM images (Figure 3) were recorded and analyzed for topographical properties of the synthesized G-AgNPs. The size of the particles was in the range of 50–70 nm. The majority of the particles were roughly spherical, with few particles showing an elongated or rod shape. In addition, most of the particles were found to be discrete in nature, while few particles were seen as aggregated, suggesting the efficacy of guggul as a capping/stabilizing agent. Furthermore, the particles showed a non-porous smooth texture without any attached foreign matters.

#### 3.4.3. Particle Size Measurement

**Figure 4:**
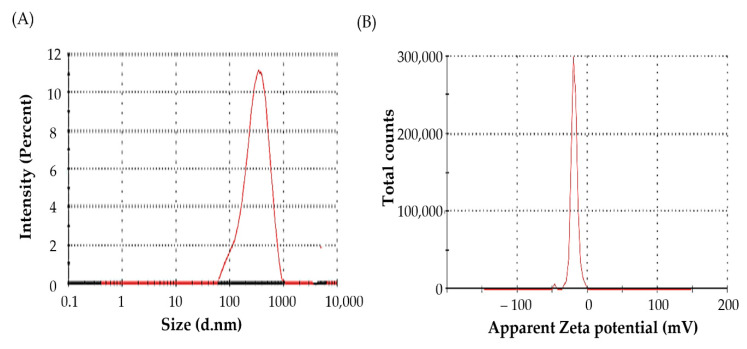
(**A**) Particle size; and (**B**) zeta potential of silver nanoparticles.

Particle size of nano-delivery systems is an essential parameter that dictates both in vitro stability and in vivo biological efficacy. Generally, smaller particles (200–400 nm) can readily extravasate from leaky vasculature, with subsequent preferential accumulation in the sites of inflammation via ELVIS effect [6]. The particle size and polydispersity index of the G-AgNPs, prepared under optimal synthesis conditions, were 337.6 ± 12.1 nm and 0.352, respectively (Figure 4A). These results suggest the propensity of the synthesized nanoparticles to be accumulated at inflammation sites via ELVIS effect.

Another essential characterization parameter of nanoparticles is the estimation of particle surface charge: zeta potential [46]. The magnitude of the zeta potential signifies the degree of electrostatic repulsion between similarly charged neighboring particles in a dispersion, which, in turn, dictates the stability of colloidal dispersions. As shown in Figure 4B, the synthesized G-AgNPs had a zeta potential of −18.9 ± 1.8 mV, indicating their stability.

#### 3.4.4. Fourier Transform Infrared (FT-IR) Analysis

**Figure 5:**
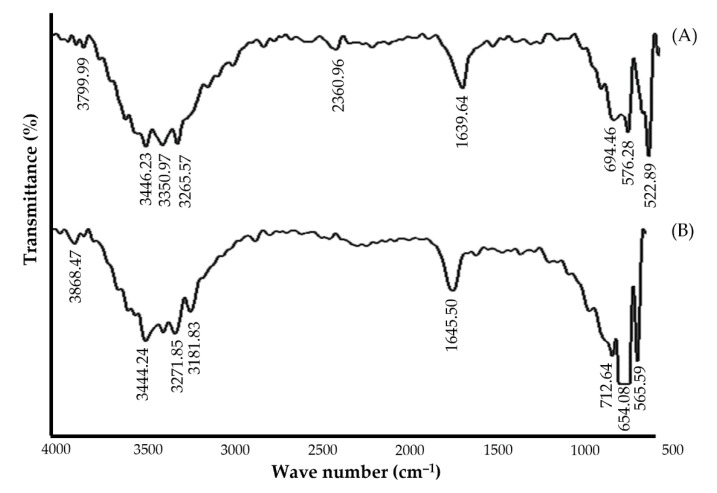
FT-IR spectra of (**A**) Guggul extract, and (**B**) Silver nanoparticles.

The FTIR spectra of guggul extract and synthesized G-AgNPs are shown in Figure 5. FTIR spectra of guggul extract show its characteristic peaks at 3444 cm^−1^ and 3272 cm^−1^ for ‒O‒H (stretching) and aromatic ‒C‒H (stretching), respectively. The peaks observed at 1645 cm^‒1^, 712 cm^−1^ and 654 cm^−1^ correspond to ‒C=O (stretching), ‒C=C (bending) and ‒C‒H (bending), respectively, as shown in Figure 5. FT-IR spectra of AgNPs exhibit peaks at 3350 cm^−1^ for ‒O‒H (stretching) and 3265 cm^−1^ for aromatic ‒C‒H (stretching), respectively. The peaks observed at 2360 cm^−1^, 1639 cm^−1^, 694 cm^−1^ and 576 cm^−1^ correspond to ‒C=C (stretching), ‒C=O (stretching), ‒C=C (bending) and ‒C‒H (bending), respectively. The alteration of peak at 1645 cm^−1^ to 1639 cm^−1^ suggests the involvement of guggul ‒C=O groups in reduction/stabilization of the synthesized AgNPs.

### 3.5. Acute Toxicity Study

Acute oral toxicity studies were conducted as per guidelines of OECD 425, and the dose was standardized. The oral administration of the silver nanoparticles at a dose of 2000 mg/kg was found to be toxic, leading to the death of the rats. A much lower dose of 1.75 mg/kg was also found to be lethal to the animals. Therefore, the dose was reduced to ~1/10th and examined at three levels (100 µg/kg, 200 µg/kg, and 300 µg/kg). It was found that these doses were non-toxic, and no mortality was observed upon oral administration for up to 7 days. Furthermore, no signs of toxicity, in terms of a reduction in body weight or physical behavior changes (depression, alterations in alertness, limb tone, grip strength, or animal gait), were observed before and after treatment. Accordingly, a test dose of 200 µg/kg was selected as a therapeutic dose of nanoparticles for testing anti-arthritic activity in rats.

### 3.6. Adjuvant-Induced Arthritis Rodent Model

In this study, female Wistar albino rats were selected for evaluating the anti-arthritic activity of synthesized AgNPs, since it is well-known that rheumatoid arthritis (RA) prevalence is more common in adult females in comparison to adult males [47]. In addition, the adjuvant-induced arthritis (AIA) model was adopted for evaluating the therapeutic efficacy of the synthesized G-AgNPs, since it resembles human RA clinical features [48]. The administration of CFA in the sub-plantar region of the right hind paw for a period of 7 days develops arthritis in animals. The arthritic score, paw volume and inflammation, and change in body weight were used as markers of the anti-arthritic effect of the silver nanoparticle throughout the term of the experiment. Furthermore, histological and biochemical evaluations were carried out post-euthanization of the rats.

#### 3.6.1. Evaluation of Anti-Arthritic Activity of AgNPs

**Figure 6:**
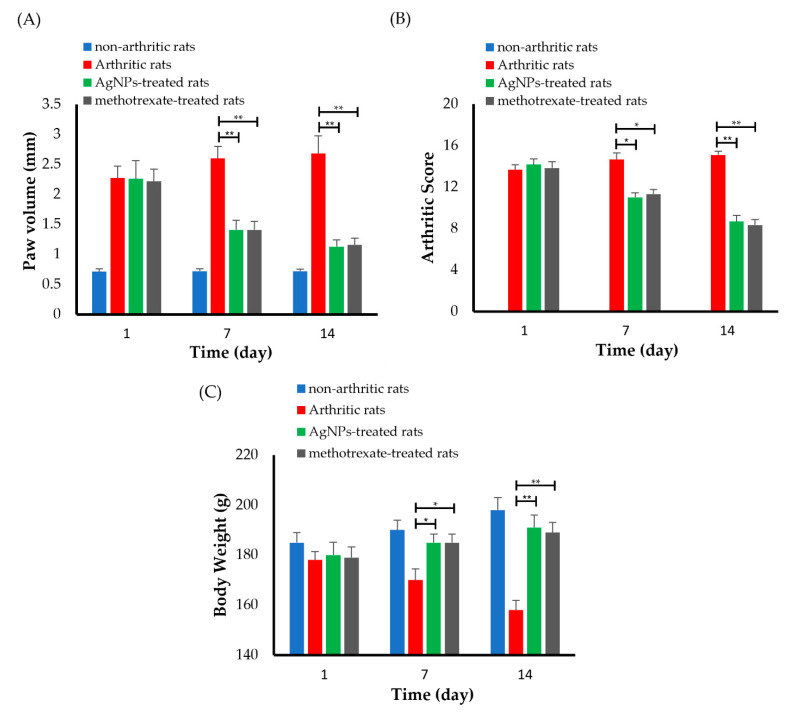
Anti-arthritic activity of silver nanoparticles. (**A**) Paw volume, (**B**) arthritic score, and (**C**) body weight of rats treated with either silver nanoparticles or a reference drug, methotrexate. * *p* < 0.5 and ** *p* < 0.01 vs. arthritic rats.

The change in paw edema has traditionally been used to measure the efficacy of an anti-inflammatory medication in RA [49]. Generally, the progression of RA in the adjuvant-induced arthritis (AIA) model can be traced by paw swelling. The decrease in paw circumference in the AIA model clearly signifies a decrease in swelling rate, which reflects the effectiveness of treatment regimen. As shown in Figure 6A, the arthritic control group showed obvious paw swelling shortly (1 day) after CFA induction, which reached high severity on day 7 and lasted for 14 days, indicating a clear sign of clinical inflammation. On the other hand, both treatment groups (G-AgNPs and methotrexate-treated groups) showed time-dependent decreases in paw volume induced by CFA. At day 14, the paw edema was significantly (*p* < 0.01) reduced by >40% upon treatment with G-AgNPs (200 µg/kg/day, p.o.), compared to the arthritic control (Figure 6A). Such a decrease in swelling rate, which might be attributed to decreased edema, attenuated inflammatory processes, and decreased synovial tissue hyperplasia [50,51]. Interestingly, the anti-inflammatory effect of AgNPs in AIA was comparable to that of the reference drug, methotrexate (0.4 mg/kg/day, i.p.). Collectively, these results suggest the potential of synthesized AgNPs in alleviating RA-related inflammation.

Evaluation of arthritic score is another important consideration in physical observation of inflammation and swelling. As shown in Figure 6B, the arthritic control rats showed the maximum arthritic score, compared to the normal control. Treatment with G-AgNPs (200 µg/kg/day, p.o.), on the other hand, significantly (*p* < 0.5) reduced the arthritic score in a treatment duration-dependent manner, compared to the arthritic control. Of note, the efficacy of G-AgNPs in reducing arthritic scores were comparable to that obtained with the reference drug, methotrexate (Figure 6B).

Another key factor to consider is the reduced food intake and subsequent body weight loss observed in experimental models. As shown in Figure 6C, arthritic control rats lost a remarkable amount of body weight and became considerably different from the other groups, presumably due to limited locomotion and/or discomfort. On the other hand, there were no significant variations in body weight between the normal non-arthritic rats and the animals receiving either G-AgNPs or the reference drug, methotrexate. These findings indicate that therapy with G-AgNPs was well tolerated and has a favorable safety profile.

#### 3.6.2. Estimation of Antioxidant Activity

**Figure 7:**
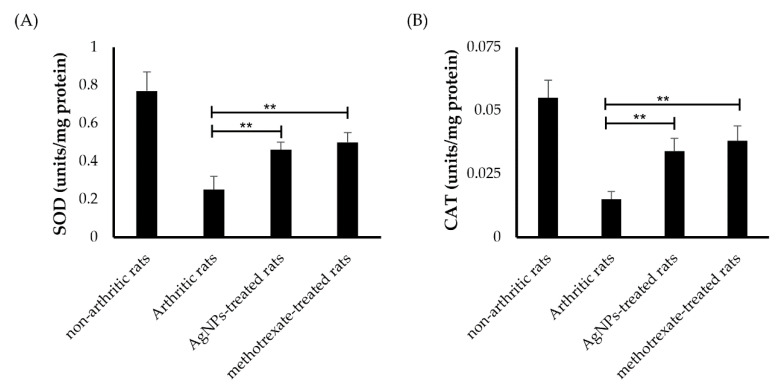
Antioxidant activity of silver nanoparticles. (**A**) Superoxide dismutase (SOD) enzyme, and (**B**) catalase (CAT) enzyme in tissue homogenates of arthritic rats treated with either silver nanoparticles or a reference drug, methotrexate. ** *p* < 0.01 vs. arthritic rats.

Besides inflammation, oxidative stress is another hallmark of RA. Many reports have emphasized the elevation of oxidative stress along with increased inflammation in patients suffering from RA [52]. Such elevation in oxidative stress has been linked to an imbalance between pro-oxidant and antioxidant defense systems [53]. Herein, the activity of two antioxidant enzymes, namely superoxide dismutase (SOD) and catalase (CAT) enzymes, was evaluated in rat paw tissue homogenate. As shown in Figure 7, the activities of SOD and catalase enzymes were reduced by 67% and 73%, respectively, in the arthritic control group, compared to the non-arthritic normal rats. On the other hand, treatment with either the synthesized G-AgNPs or reference drug (methotrexate) significantly increased (*p* < 0.05) the activities of these antioxidant enzymes compared with arthritic rats. The enzymatic activities of SOD and CAT enzymes were 1.85- and 2-folds higher in the G-AgNPs-treated group, compared to the arthritic control group (Figure 7). These results suggest that G-AgNPs could exert their anti-rheumatic activity via not only reducing RA-associated inflammation, but via restoring the pro-oxidant and antioxidant balance as well.

#### 3.6.3. Radiological Assay

**Figure 8:**
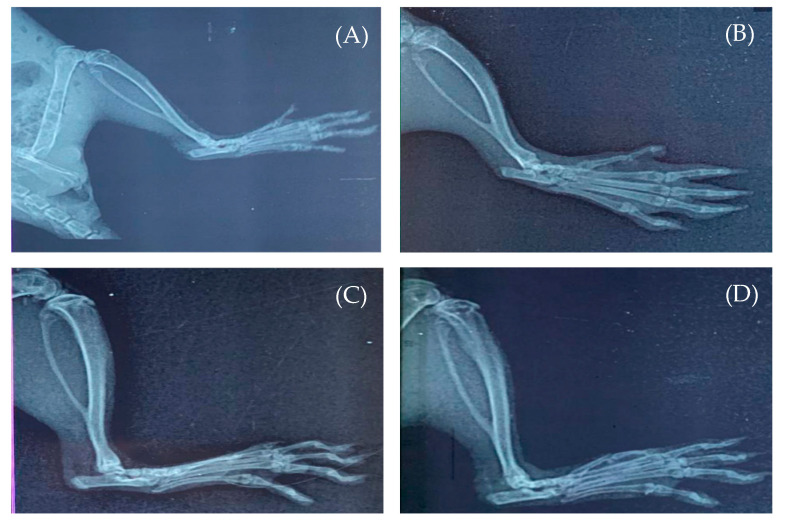
Radiological images of the right ankle joints of (**A**) normal control rats; (**B**) arthritic rats; (**C**) silver nanoparticle-treated rats; and (**D**) methotrexate-treated rats.

The radiographic examination of ankle joints of all groups of rats are depicted in Figure 8. Compared to the normal control group (Figure 8A), adjuvant-treated rats (arthritic control) showed remarkable soft tissue swelling, uneven joint spaces, and excessive bone degeneration (Figure 8B). In contrast, rats treated with G-AgNPs displayed mild to moderate tissue swelling and mild degenerative changes in the rat ankle joint (Figure 8C). Similarly, rats treated with methotrexate showed little degenerative alterations in the ankle joint, with mild edema in the surrounding tissues (Figure 8D). These results depict that G-AgNPs can successfully reverse disease progression in arthritic rats.

#### 3.6.4. Histopathology Assessment

**Figure 9:**
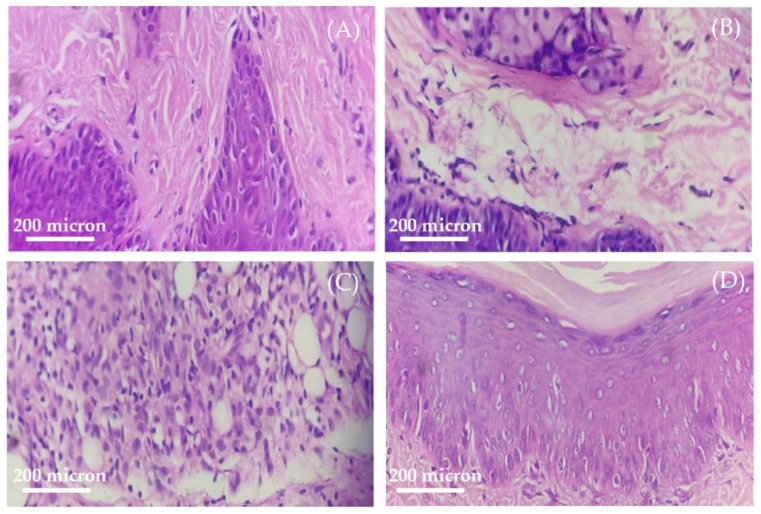
Histopathology of the right paw tissues of (**A**) normal control rats; (**B**) arthritic rats; (**C**) silver nanoparticle-treated rats; and (**D**) methotrexate-treated rats.

Aside from biochemical evidence, joint histological staining was conducted to ensure the effectiveness of treatment with G-AgNPs (Figure 9). No gross anomalies were detected in the morphology of the joint tissues of the non-arthritic control rats, whereas histopathological examinations disclosed that arthritic rats showed remarkable signs of cellular infiltration, synovial hyperplasia, and bone destruction. Treatment with G-AgNPs, on the other hand, considerably reduced joint damage and exerted only a mild degree of cellular infiltration, hyperplasia, and bone destruction. Similarly, treatment with the reference drug, methotrexate, remarkably alleviated joint tissue inflammation, as manifested by mild hyperplasia in synovial membrane and mild grade of infiltration of inflammatory cells. Collectively, this study clearly emphasized the efficacy of the synthesized G-AgNPs in suppressing inflammation inside tissues and preventing arthritis-related paw edema.

## 4. Conclusions

Guggul is a resin extract that has been used in Ayurvedic medicine for the treatment of various conditions, including arthritis. In addition, the oleo-gum resin of Guggul is rich in phytoconstituents, such as terpenoids, steroids, and lignans, which can act as powerful reducing agents. In this study, we exploited the reducing potential, along with the anti-inflammatory efficacy, of guggul for the synthesis of biostable G-AgNPs for the management of rheumatoid arthritis. The synthesized nanoparticles showed good in vitro colloidal stability and good in vivo safety profile upon oral administration. In addition, in Complete Freund’s adjuvan-induced arthritis model, the biosynthesized G-AgNPs exerted potent anti-arthritic potential, as depicted by a considerable reduction in paw thickness, as well as a lower arthritic score. Interestingly, such anti-arthritic efficacy was comparable to that of a reference standard, methotrexate. Collectively, this study is the first to report the use of biosynthesized bio-active nanoparticles for rheumatoid arthritis treatment without the addition of any medicines, and it underlines the potential use of G-AgNPs for targeting rheumatoid arthritis.

## Data Availability

Not applicable.

## Supplementary Materials

The following supporting information can be downloaded at: https://www.mdpi.com/article/10.3390/pharmaceutics14112318/s1, Table S1. Phytochemical analysis of *Commiphora mukul* (Guggul) extract.
