# Supplementary material for: Biosynthesis of Silver Nanoparticles Using Commiphora mukul Extract: Evaluation of Anti-Arthritic Activity in Adjuvant-Induced Arthritis Rat Model
Source: Pharmaceutics. 2022 Oct 28;14(11):2318. doi: 10.3390/pharmaceutics14112318 (PMC9693186; doi:10.3390/pharmaceutics14112318)
Supplement: Supplementary file 1 [file pharmaceutics-14-02318-s001.zip › pharmaceutics-1967877-supplementary.pdf]

Table S1. Phytochemical analysis of *Commiphora mukul.*

Phytochemical constituent	Test name	Observation	Inference
Alkaloids	Wagner Test	Pale yellow	– ve
Flavonoid	Lead acetate Test	White ppt.	– ve
Terpenoids	Salkowski Test	Reddish brown	+ ve
Steroids	Liebermann-Burchard’s test	Bluish green	+ ve
Carbohydrate	Molish test	Clear solution	– ve
Tannin	Ferric Chloride test	Yellow color	– ve
